# Kinetic measurements of hand motor impairments after mild to moderate stroke using grip control tasks

**DOI:** 10.1186/1743-0003-11-84

**Published:** 2014-05-11

**Authors:** Yu Ye, Le Ma, Tiebin Yan, Huihua Liu, Xijun Wei, Rong Song

**Affiliations:** 1 School of Engineering, Sun Yat-sen University, Guangzhou, PR China; 2 Department of Rehabilitation Medicine, Sun Yat-sen Memorial Hospital, Sun Yat-sen University, Guangzhou, PR China

**Keywords:** Upper extremity, Movement disorders, Stroke, Rehabilitation

## Abstract

**Background:**

The aim of this study is to investigate quantitative outcome measurements of hand motor performance for subjects after mild to moderate stroke using grip control tasks and characterize abnormal flexion synergy of upper extremities after stroke.

**Methods:**

A customized dynamometer with force sensors was used to measure grip force and calculate rotation torque during the sub-maximal grip control tasks. The paretic and nonpartic sides of eleven subjects after stroke and the dominant sides of ten healthy persons were tested. Their maximal voluntary grip force was measured and used to set sub-maximal grip control tasks at three different target force levels. Force control ability was characterized by the maximal grip force, mean force percentage, coefficient of variation (CV), target deviation ratio (TDR), and rotation torque ratio (RTR). The motor impairments of subjects after stroke were also evaluated using the Fugl-Meyer assessment for upper extremity (FMA-UE) and Wolf Motor Function Test (WMFT).

**Results:**

Maximal grip force of the paretic side was significantly reduced as compared to the nonparetic side and the healthy group, while the difference of maximal grip force between the nonparetic side and the healthy group was not significant. TDR and RTR increased for all three groups with increasing target force level. There were significant differences of CV, TDR and RTR between the paretic side and the healthy group at all the force levels. CV, TDR and RTR showed significant negative correlations with FMA-UE and WMFT at 50% of maximum grip force.

**Conclusions:**

This study designed a customized dynamometer together with an innovative measurement, RTR, to investigate the hand motor performance of subjects after mild to moderate stroke during force control tasks. And stroke-induced abnormal flexion synergy of wrist and finger muscles could be characterized by RTR. This study also identified a set of kinetic parameters which can be applied to quantitatively assess the hand motor function of subjects after mild to moderate stroke.

## Background

Post-stroke impairments in motor control can cause functional limitations in the activities of daily living (ADL), for example in grasping and object manipulation [1,2]. Compared with healthy persons, stroke survivors usually have a lower quality of life due to such functional limitations [3]. Therefore, a major concern for patients after stroke is recovery of upper extremity motor function [4]. Patients after stroke can regain motor functions and get back to near-normal ADL performance through rehabilitation [5]. Therapists devise therapeutic interventions based on the motor status of each patient [6]. The motor deficits are often evaluated using clinical scales such as the Action Research Arm Test and the Fugl-Meyer assessment (FMA) [7-9]. However, these semi-quantitative clinical scales lack reproducibility and may not be sensitive enough to monitor changes caused by stroke and during rehabilitation [10]. Objective and specific information about motor function impairments is needed during rehabilitation.

Recent quantitative investigations of hand motor function can be summed up into four categories. First, finger movements [11,12] could reflect precise hand motor functions. Most subjects after stroke can hardly control finger movements independently and flexibly [13] and the analysis of finger movements are often combined with grip force control. Secondly, gripping to counteract a physical load is generally studied during stationary holding [14,15]. Static gripping movements are particularly well suited for investigating the coupling between grip force and the load [16]. Thirdly, dynamic gripping movements [16-18] are commonly used to estimate upper extremity motor function after stroke. Last, power grip reflects force generating capacity, and is often tested before other grip control tasks are assessed. Previous studies also stated that power grip force not only reflected the force generation capacity, but also required careful control when sustaining force at a certain level [19-21]. Lindberg et al. designed a grip force tracking task to analyse grip force modulation [22]. Grip control should be considered an important way for screening subjects after stroke in clinical settings [23]. Abnormal muscle synergies are often found in patients after stroke, which seriously influences motor function of paretic upper extremities [15,24], while flexion synergy of the upper extremity muscles has seldom been investigated.

During grip control tasks, the activation of flexor digitorum muscles is needed, while the abnormal muscle synergy might cause the activation of neighboring flexors, flexor carpi muscles, and result in the involuntary flexion of wrist in patients after stroke. In this study, the customized device can measure the grip force and rotation torque, which reflect the activations of two neighbouring flexors: flexor digitorum muscles and flexor carpi muscles, respectively. Rotation torque ratio (RTR) was proposed in this study to characterize the mutual effects of the two neighbouring flexors during grip control tasks. RTR was defined with the rotation torque divided by grip force to identify the relative value of abnormal muscle synergies between wrist and finger. The kinetic parameters (maximal grip force, mean force percentage, CV, TDR, and RTR) in the paretic and nonparetic side of the subjects after stroke were compared with those of healthy persons to quantify stroke-induced discrimination. Correlations were addressed between clinical scales and the kinetic parameters observed on the paretic side of subjects after stroke to determine the relationship between the degree of motor impairment and these parameters.

## Methods

### Participants

Two groups of subjects were recruited in this study. They were eleven subjects after stroke (mean age: 54 ± 15.84 years, three females, eight males) and ten age-matched healthy persons (mean age: 51.7 ± 6.24 years, five females, five males). Table 1 summarized the basic clinical information of subjects after stroke. The subject selection criteria included: (1) hemiparesis resulting from a single unilateral lesion of the brain with onset at least one month prior to data collection; (2) able to generate voluntary contractions of the both hands; (3) moderate and mild stroke scoring more than 33 on Fugl-Meyer assessment for upper extremity (FMA-UE) [25]; (4) no visual, cognitive or attention defect which prevented following the experimental procedures as indicated by a score of 23 or more on the mini mental state examination (MMSE) [26]. In the healthy group, all the participants were right-handed. Informed consent was obtained from all participants. The study was approved by the Ethics Committee of Sun Yat-sen University.

**Table 1:** Background data of the subjects after stroke

**Subject**	**Sex**	**Age (year)**	**Duration (month)**	**Paretic hemisphere**	**Assessment scale**			
					**FMA-UE (0–66)**	**WMFT (0–75)**	**MMSE (0–30)**	**MAS (0–6)**
1	F	63	3	R	61	62	30	1
2	M	40	4	R	63	67	30	1
3	M	22	1.5	R	51	50	30	1
4	F	52	2.5	L	64	71	30	0
5	M	73	1	L	48	45	30	0
6	M	64	2.5	L	60	57	30	0
7	M	49	2	R	65	73	30	0
8	M	72	6	R	65	72	30	0
9	F	63	5	R	42	46	30	0
10	M	59	4.5	L	56	60	30	1
11	M	37	2	L	46	48	30	3

*Abbreviations:R* right, *L* left, *FMA-UE* Fugl-Meyer Assessment for Upper Extremity, *WMFT* Wolf Motor Function Test, *MMSE* Mini-Mental State Examination, *MAS* Modified Ashworth Scale.

Hand and arm motor impairments of the subjects after stroke were assessed using both FMA-UE [9,25] and WMFT [27]. Muscle tone in the upper extremities was evaluated using the modified Ashworth Scale [28].

### Apparatus

**Figure 1:**
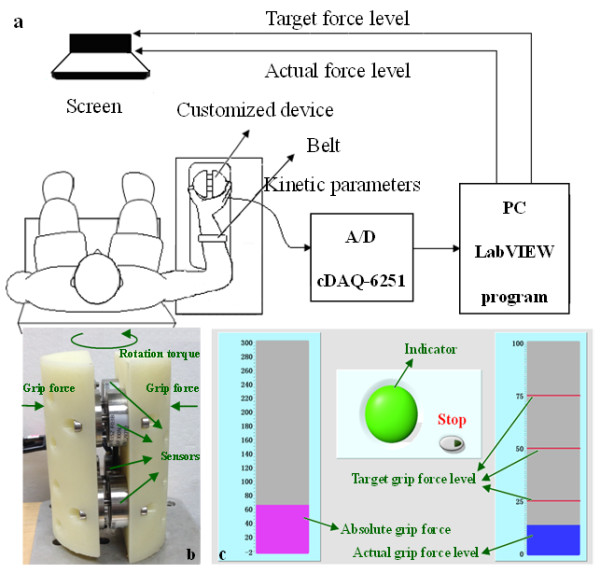
**Illustration of the experiment. (a)** Schematic diagram of the experimental manipulation; **(b)** the dynamometer; **(c)** the LabVIEW interface for the tasks.

As showed in Figure 1b, the customized grip dynamometer was cylindrical with a diameter of 60 mm and a height of 90 mm, and it was fixed to the experiment table. The dynamometer contained four force sensors (LSZ-F03B, Suzhou Battelle Automation Equipment Company, Suzhou, China) placed axisymmetrically (horizontal centre-to-centre distance: 28 mm, vertical centre-to-centre distance: 40 mm) to measure the grip force (0-200 N, with a precision of ±0.001 N) and calculate the rotation torque (up to ±80 Nm, precision ±0.01 Nm).

A 16-bit analogue to digital converter (cDAQ-6251, National Instruments, Austin, Texas, USA) sampled the force data at a rate of 1000 Hz. A computer screen in front of the participants displayed both the target and the actual force levels. Figure 1c shows the visual feedback viewed by each participant. The three stationary horizontal red lines represented the three target force levels, and the movable horizontal blue bar represented the actual force level produced by the participant in real time. A customized LabVIEW program (LabVIEW 2011, National Instruments Corporation, Austin, Texas, USA) controlled the visual presentation of each task, and it also processed data during task performance. The force and torque data were saved for offline analysis.

### Procedure

Both the paretic and nonparetic sides of the stroke group and the dominant sides of the healthy group were tested. The participants were instructed to sit with their backs straight against the backrest of the chair and rest the arm to be tested on the tabletop. The upper arm was in a neutral adducted position with approximately 15-20° of shoulder flexion and 90° of elbow flexion. The dynamometer was grasped with the thumb and the four fingers in opposition. During testing, forearm of each participant was constrained by a belt to standardize grip position and prevent forearm motion (Figure 1a).

Each participant was first instructed to apply maximal grip force (MGF) to the dynamometer three times, holding it for 5 seconds each time while an indicator light was illuminated. The largest MGF among the three measurements was used to normalize the grip force for the sub-maximal force level tasks. The participants were then instructed to apply sub-maximal force sufficient to position the blue bar at the red target line and hold it for 5 s, which was in accordance with previous work [29]. The targets were 25%, 50% and 75% of the MGF, which were arranged in a random sequence. A rest period of 30 s was provided between each trial to minimize fatigue. Each force target was presented three times for a total of 9 sub-maximal trials with each participant.

### Data analysis

A fourth-order Butterworth low-pass filter with a cut off frequency of 20 Hz was used to filter the force signals. The force data were also trimmed by 1 s at the beginning and 1 s at the end of the trial to account for initial force adjustments and terminal force changes caused by the anticipation of trial’s end. All the data analysis was accomplished using the Matlab signal processing toolbox (Matlab R2009b, MathWorks, Natick, Massachusetts, USA).

Motor performance of each participant was evaluated in terms of mean force percentage, coefficient of variation (CV), target deviation ratio (TDR), and the RTR of the force output.

Mean force percentage was calculated to identify the degree of task completion:

(1) $$\mathrm{Mean}\;\mathrm{Force}\;\mathrm{Percentage}=\frac{\mathrm{Mean}\;\mathrm{Force}}{\mathrm{Maximal}\;\mathrm{Grip}\;\mathrm{Force}}\times 100$$

This study calculated coefficient of variation (CV) to measure the relative variability of force production during each task. CV was the result of dividing standard deviation of force data by the mean force:

(2) $$\mathrm{CV}=\frac{\mathrm{Standard}\;\mathrm{Deviation}}{\mathrm{Mean}\;\mathrm{Grip}\;\mathrm{Force}}$$

TDR assessed the subjects control in maintaining grip force. It was the mean deviation from the desired force level computed as:

(3) $$\mathrm{TDR}=\frac{\sqrt{\frac{1}{N}\sum_{i=1}^{N}{\left(p\left(i\right)-p_{0}\right)}^{2}}}{p_{0}}$$

where N was the total number of samples, *p*_0_ was the target force and *p*(*i*) was the actual grip force at ith sampling.

To investigate flexion synergy of wrist and finger muscles during grip control tasks, RTR was calculated by calculating the ratio of rotation torque to actual grip force during each task. The total rotation torque was the sum of torques registered by the four sensors, and it was calculated as below:

(4) $$\mathrm{RTR}=\frac{\sum_{j=1}^{4}F_{j}\times L_{j}}{\sum_{j=1}^{4}F_{j}}$$

where $\sum_{j=1}^{4}F_{j}\times L_{j}$ is the net rotation torque of the four sensors, $p=\sum_{j=1}^{4}F_{j}$ is the net actual grip force. Here, *F*_*j*_ is the grip force from the jth sensor, and *L*_*j*_ is the distance between the centre of the jth sensor and the axis of the dynamometer.

These outcome variables were subjected to two-way analysis of variance (ANOVA) assuming two main factors: one factor was group (paretic side, nonparetic side, or healthy group) and the other was the target force level (25%, 50% or 75% of the MGF). The statistical model assessed the main effects relating the two factors with the observations. The ANOVA results were adjusted using a Bonferroni post hoc test. Pearson correlation coefficients were computed to find the relationship between the clinical scales, including FMA and WMFT, and the above kinetic parameters. All the statistical tests were conducted with the significant level set at 0.05. All statistical work was performed with the aid of version 19 of the SPSS software package (SPSS Inc, Chicago, USA).

## Results

**Figure 2:**
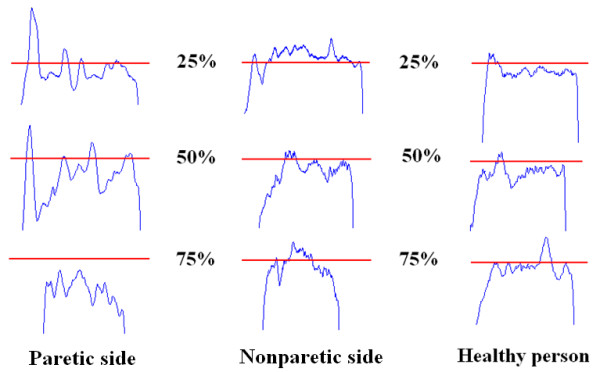
**Example of isometric force output at 25%, 50% and 75% of MGF.** Examples from the paretic and nonparetic side of a subject after stroke and a healthy person. The red line displays the target force level and the blue line indicates the force level produced by the participant.

MGF varied among the three groups from 17.68 to 142 N on the paretic side, 92 to 271.22 N on the nonparetic side, and 110 to 346 N in the control group. The average MGF for the paretic side of the stroke participants (83.1 ± 10.89 N) was significantly less than that of the nonparetic side (155.02 ± 16.2 N; p < 0.01) and that of the healthy participants (184.46 ± 18.52 N; p < 0.01). There was larger MGF in the healthy group than that of the nonparetic side, but the difference of MGF between the nonparetic side and healthy group was not significant. Figure 2 showed a typical example of the force outputs at the 25%, 50%, and 75% force levels generated by one healthy participant and both sides of one stroke participant. By analyzing force data of the two participants, the force output of the paretic side showed greater fluctuations than the other two at the same force level. The largest discrepancy occurred between the 75% force level and the actual force output on the paretic side.

### Variability and accuracy of force control

**Figure 3:**
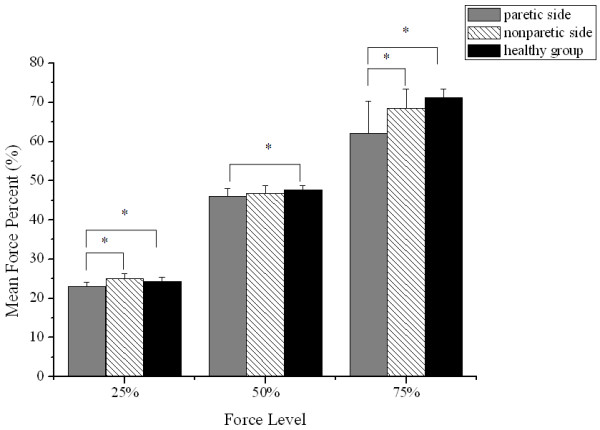
**Comparison of mean force percentages.** Mean force percentages from the paretic and nonparetic sides of the stroke group and from the healthy group at 25%, 50% and 75% of MGF. Vertical bars indicate the SD (*p < 0.05).

Figure 3 showed the overall mean force percentages of the three groups: the paretic side (25%: 22.96 ± 1.07, 50%: 46.06 ± 2.04, 75%: 62.05 ± 8.31), the nonparetic side (25%: 25.09 ± 1.31, 50%: 46.82 ± 1.95, 75%: 68.47 ± 4.86) and the healthy group (25: 24.26 ± 1.24, 50%: 47.61 ± 1.13, 75%: 71.18 ± 2.19). Post hoc analysis confirmed that the paretic side of the stroke group generated significantly less mean force percentage than the healthy group at all three target force levels (25%: p = 0.019; 50%: p = 0.047; 75%: p < 0.01). Within the subjects after stroke, the paretic side generated significantly lower mean force percentages than the nonparetic side at the 25% (p < 0. 01) and 75% force level (p = 0.038). There was no significant difference in mean force percentage between the nonparetic side and the healthy group at all levels.

**Figure 4:**
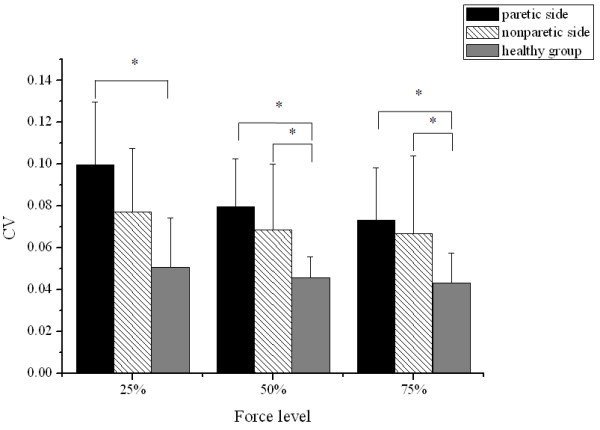
**Comparison of CV.** CV from the paretic and nonparetic sides of the stroke group and from the healthy group at 25%, 50% and 75% of MGF. Vertical bars indicate the SD (*p < 0.05).

Based on two-way ANOVA, the effects of both group and force level on CV were significant (p < 0.01). As shown in Figure 4, there were significantly larger CV on the paretic side at the three levels (25%: p = 0.049; 50%: p < 0.01; 75%: p < 0. 01) and the nonparetic side at the 50%, 75% level (50%: p = 0.041; 75%: p = 0.038) as compared to the healthy group. Moreover, the largest value of CV was found at the 25% force level.

**Figure 5:**
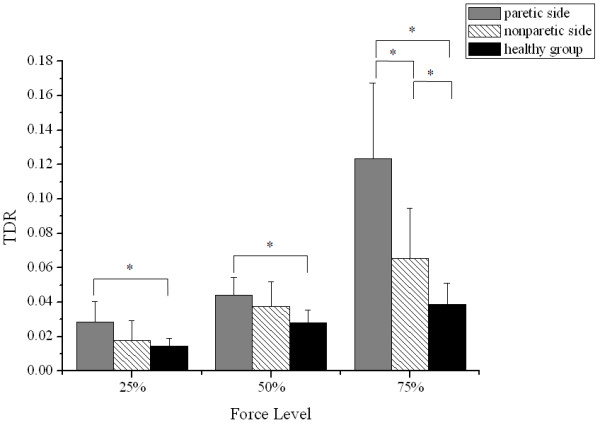
**Comparison of TDR.** TDR from the paretic and nonparetic sides of the stroke group and from the healthy group at 25%, 50% and 75% of MGF. Vertical bars indicate the SD (*p < 0.05).

Figure 5 displayed the mean TDR values of the three groups across all sub-maximal force levels. There was a monotonic increase in TDR for the three groups as the target force level increased. Two-way ANOVA showed significant relationship with TDR for both the sub-maximal force level (p < 0.01) and the group main effect (p < 0.01). Post hoc analysis revealed significantly larger TDR on the paretic side at the three levels (25%: p < 0.01; 50%: p < 0.01; 75%: p < 0. 01) and significantly larger TDR on the nonparetic side at the 75% level (p = 0.013) as compared to the healthy group. The difference between the two sides of the stroke group was significant only at the 75% force level (p < 0.01).

### Rotation torque ratio

**Figure 6:**
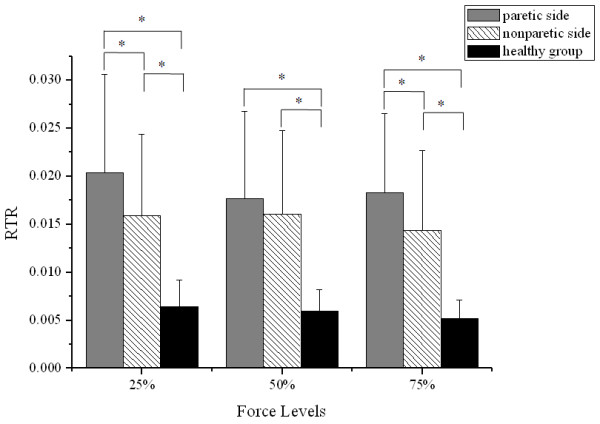
**Comparison of RTR.** RTR from the paretic and nonparetic sides of the stroke group and from the healthy group at 25%, 50% and 75% of MGF. Vertical bars indicate the SD (*p < 0.05).

Figure 6 showed that the mean RTR of both sides of the stroke group had a larger standard deviation than that of the healthy group at all force levels. Two-way ANOVA revealed that the group effect on RTR was highly significant (p < 0.01). However, no significant relationship between sub-maximal force level and RTR was observed. Post hoc analysis showed that both the paretic side and the nonparetic side of the stroke group had significantly larger RTR than the healthy group at all force levels (each p < 0.01). The RTR values of the paretic side were significant larger than those of the nonparetic side at the 25% and 75% force levels (25%: p = 0.048; 75%: p = 0.039).

### Relationship between kinetic parameters and clinical scales

Pearson’s correlation coefficients relating the measures on the paretic side of the stroke group and the clinical scales were displayed in Tables 2 and 3. There was no significant correlation between mean force percentage and the clinical scales at any force level. CV, TDR and RTR were significantly negatively correlated with FMA at the 25% and 50% force levels. The significant correlation between CV and WMFT was found at the 50% level. The correlation between TDR and WMFT results was significant at the 25% and 50% force levels, and there was also significant correlation between RTR and WMFT at all force levels.

**Table 2:** Correlation (r) of parameters in the paretic side and FMA with related probability (p)

**Force measurements**	**25% MVC**		**50% MVC**		**75% MVC**	
	**r**	**p**	**r**	**p**	**r**	**p**
**Mean force percent**	0.475	0.14	0.451	0.164	0.44	0.176
**CV**	-0.712*	0.014	-0.722*	0.012	-0.401	0.221
**TDR**	-0.868**	0.001	-0.775**	0.005	-0.497	0.12
**DTR**	-0.882**	<0.001	-0.786**	0.04	-0.583	0.06

*p < 0.05.

**p < 0.01.

**Table 3:** Correlation (r) of parameters in the paretic side and WMFT with related probability (p)

**Force measurements**	**25% MVC**		**50% MVC**		**75% MVC**	
	**r**	**p**	**r**	**p**	**r**	**p**
**Mean force percent**	0.464	0.15	0.411	0.209	0.362	0.275
**CV**	-0.591	0.056	-0.739**	0.009	-0.506	0.112
**TDR**	-0.819**	0.002	-0.651*	0.03	-0.39	0.235
**DTR**	-0.713*	0.014	-0.684*	0.02	-0.543	0.084

*p < 0.05.

**p < 0.01.

## Discussion

Hand motor function between subjects after mild to moderate stroke and age-matched healthy persons was compared in terms of mean force percent, CV, TDR, and RTR during grip control tasks in this study. Kinetic analysis has been used as a reliable measure of impaired upper limb motor function in subjects after stroke [30]. CV and TDR could reflect variability and accuracy of grip force control, respectively, and RTR was applied to characterize abnormal flexion synergy. This study investigated a clinically feasible protocol for quantitatively assessing hand motor function after mild to moderate stroke using grip control tasks.

### Variability and accuracy of force control

The smallest force amplitude was found on the paretic side. The decreased amplitude reflected the decrease in force production capacity of the upper extremity muscles normally observed after stroke [31]. Stroke-induced damages in the corticospinal system [32] and muscle atrophy caused by long-term disuse [33] may account for the reduced amplitude of force production and the decreased accuracy displayed by the subjects after stroke. Previous study reported that subjects after stroke and healthy participants performed tasks at 10%, 20%, and 30% of maximal voluntary contraction [22]. Force control tasks were performed at 5%, 25%, and 50% of maximal voluntary contraction to study the differences in upper extremity motor function between stroke survivors and healthy persons [34,35]. The present tasks included not only mild to moderate force levels but also a higher force level which assessed upper extremity motor function over a wider range. The effect of force level to kinetic measurements can also be investigated, and the force level with less variability in the measures and stronger correlation between the measures and clinical scales can be selected for future clinical use.

Stroke-induced impairments in force control ability also caused the increased variability of force control [35]. Increased motor variability after stroke is related with multiple structural and functional changes in the peripheral and central nervous systems, such as fluctuations in the motor unit discharge rate [36]. Increased motor output fluctuations restricted the ability of an individual to exert a steady trajectory [37] or move upper extremity accurately to the target [38]. In reverse, a pervious study reported that improvements in muscle strength would improve force control ability after strength training of upper extremities for poststroke patients [39].

Previous studies have found that subjects after stroke performed elbow extension and flexion movements with greater error compared to the healthy subjects [40,41]. Mercier and Bourbonnais used relative strength which was normalized twice to compare the difference between muscle groups [42]. In this study, accuracy of force production was represented by the mean force percentage and TDR, which are an absolute value and a relative value, and the results of the two parameters were consistent with those studies [40-42]. An increase in motor variability could also result in a reduction in the accuracy of force production.

### Rotation torque ratio

Undamaged brain stem pathways are activated by reduced cortical inhibition after corticospinal injury. This induces abnormal muscle contraction patterns [43,44]. Neural coupling after stroke reduces the ability to control the upper extremity joints independently [45], and flexion synergy is a common movement pattern in chronic stroke [24]. Krabben et al. measured the synergistic movement patterns using shoulder and elbow angle sensors integrated with a robotic exoskeleton during a circle drawing task [46]. Kung et al. designed a series of reaching tasks to explore the abnormal synergies of subjects after stroke [47]. A previous study developed a new sensing device for detecting abnormal symptoms of upper extremities for subjects after stroke using grip rotation angle [48]. The significant differences of RTR between the paretic side and healthy group confirmed its feasibility in characterizing abnormal synergy of upper extremities after stroke. Moreover, RTR was independent of the effect of force levels which indicated its stability during measurement of flexion synergy.

### Relationship between kinetic parameters and clinical scales

The significant negative correlation between clinical scales and the two parameters (CV and TDR) at the 25% and 50% force level showed its relationship with clinical scales in quantitative evaluation of motor function for subjects after stroke. This study considered the findings at the 75% force level as representing high force level while the 25% and 50% force level are more typical of the force levels involved in functional activities [49]. And at the 75% force level, all these parameters showed larger variation than at the other two levels. That might explain why no significant correlation between the parameters and the clinical scales was found at the 75% force level. WMFT provides insight into joint-specific and total limb movements to properly assess synergy patterns [27]. The significant correlations between RTR and WMFT were consistent across all force levels, which could reflect the potential of RTR as a measurement to characterize flexion synergy of upper extremities after stroke.

Clinical scales used as measurements of recovery for subjects with mild motor impairment are limited by a ceiling effect [25]. The significant correlations found between the clinical scales and kinetic parameters suggested that the kinetic parameters studied here could be useful for quantitatively evaluating hand motor function after mild to moderate stroke using grip control tasks. They might potentially be applied in clinic as a useful complement to clinical scales.

### Study limitations

Limitations of this study should be addressed when interpreting the results. All the subjects were in the 1-to-6-month span after stroke onset. This is consistent with the practice of previous studies [50,51]. In the future work, a large number of stroke patients in chronic stage should also be investigated in order to further confirm the study. After the optimal setting and method are validated, a user-friendly interface and easy-to-use instructions will be needed to facilitate the application in clinic.

## Conclusions

This study designed a customized dynamometer and used it to investigate the hand motor performance of subjects after mild to moderate stroke during force control tasks. The RTR provided a new measurement which could characterize abnormal flexion synergy of wrist and finger muscles after stroke. The results indicated that grip control tasks with various force levels have potential for quantitatively evaluating hand motor function after mild to moderate stroke.

## Abbreviations

ADL: Activities of daily living; FMA: Fugl-Meyer assessment; FMA-UE: Fugl-Meyer assessment for upper extremity; MMSE: Mini-Mental State Examination; WMFT: Wolf Motor Function Test; MGF: Maximal grip force; CV: Coefficient of variation; TDR: Target deviation ratio; RTR: Rotation torque ratio; ANOVA: Analysis of variance.

## Competing interests

The authors declare that they have no conflict of interest in publishing these results.

## Authors’ contributions

YY designed the study and carried out the experiments. TBY, LM, HHL and XJW helped to carry out the experiments. YY and RS analysed the data, interpreted the results, and drafted and revised the manuscript. All the authors approved the final version of the manuscript.
